# Neonatal intra-atrial baffle repair for isolated ventricular inversion with left isomerism: a case report

**DOI:** 10.1186/s40792-020-01016-3

**Published:** 2020-09-29

**Authors:** Yuta Kuwahara, Yukihiro Takahashi, Yuya Komori, Naohiro Kabuto, Naoki Wada

**Affiliations:** Department of Cardiovascular Surgery, Sakakibara Heart Institute, 3-16-1, Asahicho, Fuchu, Tokyo 183-0003 Japan

**Keywords:** Isolated ventricular inversion, Neonate, Intra-atrial baffle repair

## Abstract

**Background:**

Discordant atrioventricular connection with concordant ventriculoarterial connection, otherwise known as isolated ventricular inversion (IVI), is an extremely rare congenital cardiac malformation. Reports on the corrective surgery for this anomaly in neonates are few, and the procedure is difficult and complicated. Herein, we report our use of atrial septostomy as a palliative procedure followed by corrective surgery for the repair of neonatal IVI with situs ambiguous(inversus) morphology.

**Case presentation:**

A 2-day-old girl weighing 3.5 kg was admitted to our hospital with a low oxygen saturation (SpO_2_) of 70% She was diagnosed with IVI [situs ambiguous(inversus), D-loop, and D-Spiral], atrial septal defect, patent ductus arteriosus (PDA), interrupted inferior vena cava with azygos continuation to the left superior vena cava (SVC), and polysplenia by transthoracic echocardiography and cardiac computed tomography. We planned to perform corrective surgery and decided to first increase interatrial mixing by performing surgical atrial septostomy and PDA ligation 7 days after birth. However, despite the surgical septostomy, pulmonary venous blood flowed toward the right ventricle via the tricuspid valve rather than toward the left-sided atrium and hypoxemia persisted. We decided to perform the intra-atrial switch procedure at the age of 17 days via a re-median sternotomy. The cardiopulmonary bypass (CPB) circuit was established with ascending aorta and venous drainage through the SVC and hepatic veins. Utilizing a left-sided atrium(l-A) approach, a bovine pericardial patch was used for the intra-atrial baffle, which was trimmed into a trouser-shaped patch. Continuous suture using the patch was lying from the front of the right-sided upper pulmonary vein and rerouted SVC, hepatic vein, and coronary sinus to the tricuspid valve. Overall, CPB weaning proceeded smoothly; however, direct current cardioversion was performed for junctional ectopic tachycardia. The postoperative course was uneventful. Her postoperative SpO_2_ improved (approximately 99–100%); overall, the patient showed clinical improvement. Discharge echocardiography showed normal biventricular function and an intact atrial baffle with no venoatrial or atrioventricular obstruction.

**Conclusion:**

We successfully performed an intra-atrial switch procedure for isolated ventricular inversion in a neonate. Long-term follow-up will be necessary to ensure the maintenance of optimal cardiac function.

## Background

Discordant atrioventricular connection with concordant ventriculoarterial connection, otherwise known as isolated ventricular inversion (IVI), is an extremely rare congenital cardiac malformation [1]. IVI results in parallel circulation, similar to transposition of the great arteries (TGA). Ventricular septal defect (VSD) is commonly associated with IVI. Without corrective surgery, survival beyond infancy is rare. Reports on the corrective surgery for this anomaly in neonates are few, and the procedure is difficult and complicated [2, 3]. Consequently, the surgical strategy for neonatal IVI remains challenging, even more so when the atrial situs is ambiguous(inversus).

Herein, we report our use of atrial septostomy as a palliative procedure followed by corrective surgery for the repair of neonatal IVI showing situs ambiguous(inversus) morphology.

## Case presentation

**Fig. 1:**
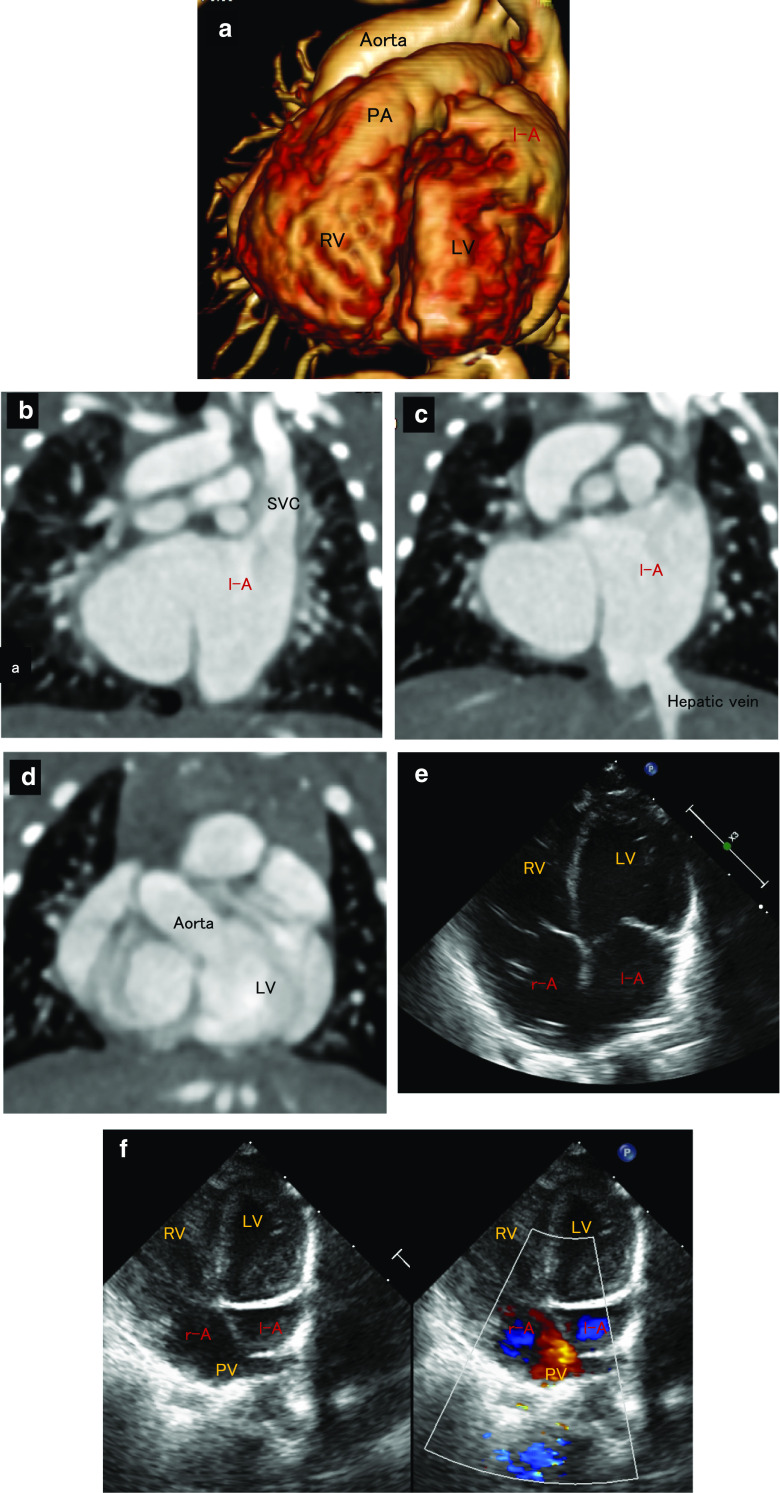
The computed tomography scans (**a**–**d**), and the transthoracic echocardiography (**e**, **f**) obtained from the patient. These figures indicate situs ambiguous(inversus), D-loop, and D-Spiral, atrioventricular discordance, ventriculoarterial concordance

A 2-day-old girl who weighed 3.5 kg was admitted to our hospital because of low oxygen saturation (SpO_2_). Her SpO_2_ was 70% and her previous doctor suspected total anomalous pulmonary venous return as the cause. She was diagnosed with IVI (situs ambiguous(inversus), D-loop, and D-Spiral), atrial septal defect (ASD), patent ductus arteriosus (PDA), interrupted inferior vena cava (IVC) with azygos continuation to the left superior vena cava (SVC), and polysplenia by transthoracic echocardiography and cardiac computed tomography (CT) (Fig. 1).

From admission, she had PDA-dependent hemodynamics, and she was started on lipo-prostaglandin E1 therapy. At the age of 5 days, she began developing congestive heart failure due to decreased pulmonary vascular resistance and the increased blood flow of the PDA. It became difficult to control her heart failure due to the PDA-dependent hemodynamics.

We believed that closure of the PDA would cause further hypoxia. We planned to perform corrective surgery, but very few case reports of an intra-atrial switch procedure for IVI in neonates are currently available. Therefore, we first considered increasing interatrial mixing, but because balloon atrial septostomy is difficult for an interrupted IVC, we performed surgical atrial septostomy and PDA ligation at the age of 7 days.

Despite the surgical septostomy, pulmonary venous blood flowed toward the right ventricle via the tricuspid valve rather than toward the left-sided atrium, and hypoxemia persisted. Various measures, such as nitric oxide administration and volume loading, were not effective for hypoxemia over several days. The SpO_2_ was around 60% and the partial pressure of arterial oxygen was around 30 mmHg, which was very low; hence, we decided to perform the intra-atrial switch procedure.

**Fig. 2:**
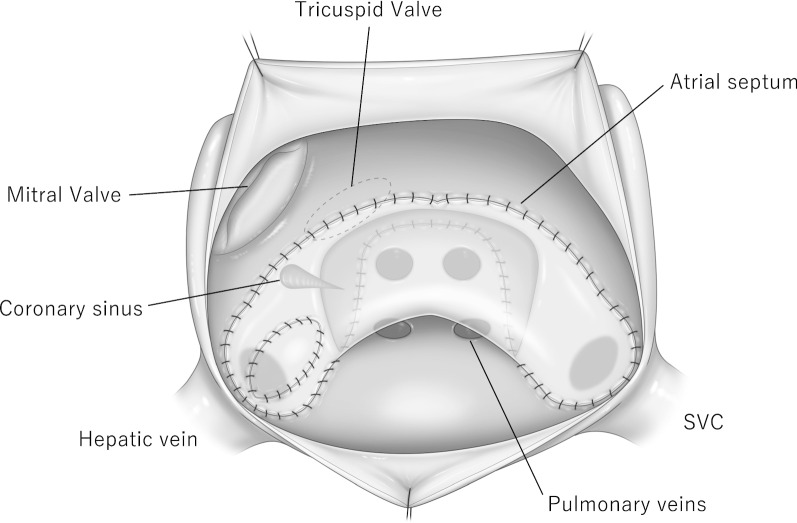
Surgical schema of intra-atrial baffle repair viewed through a left-sided atriotomy

**Fig. 3:**
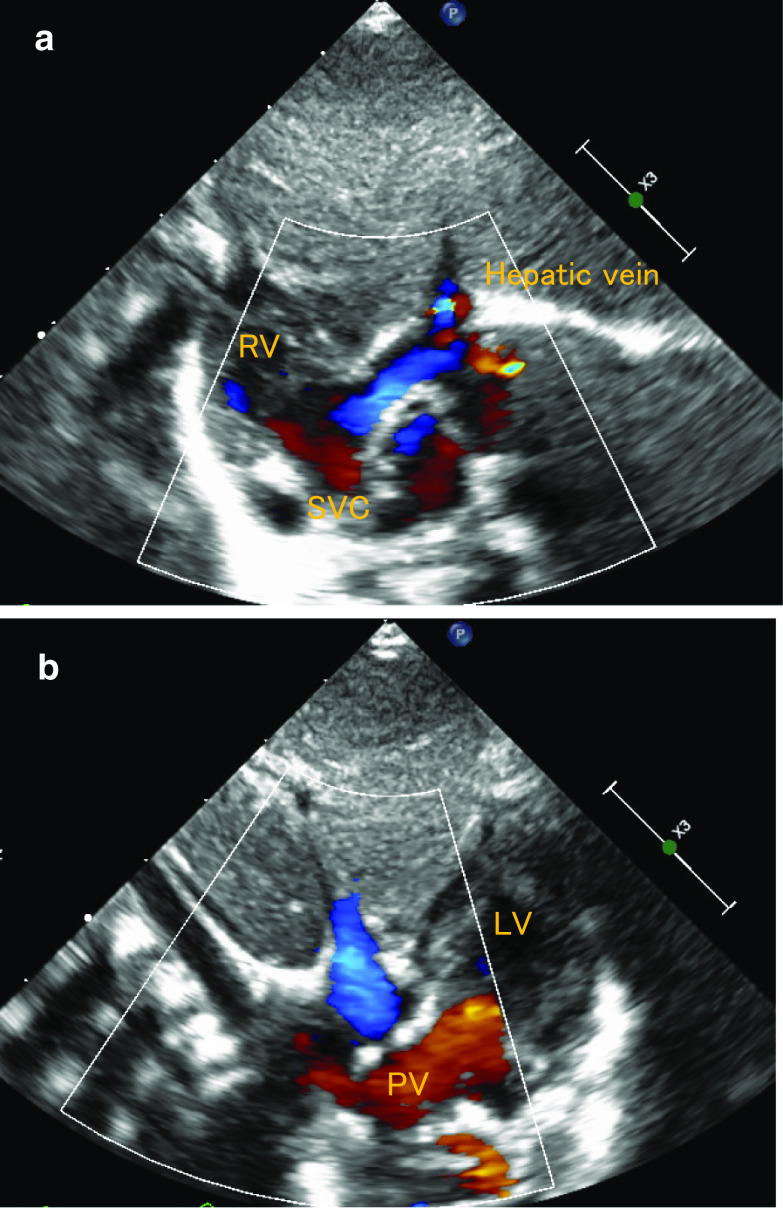
**a**, **b** The transthoracic echocardiography taken after Mustard operation showed that the systemic venous flow routed to the right-sided atrium through the systemic venous baffle, whereas the pulmonary venous flow was routed to l-A through the pulmonary venous baffle

The surgery was carried out at the age of 17 days via a re-median sternotomy. The cardiopulmonary bypass (CPB) circuit was established with ascending aorta and venous drainage through the SVC and hepatic veins. Myocardial protection was provided with hypothermia and the administration of antegrade cold blood glucose-insulin-potassium solution. A left-sided atrium(l-A) approach was employed and sufficient enlargement of the ASD was confirmed. Actually, both atria are left atrium in this case. The coronary sinus (CS) was cut back about 10 mm. A bovine pericardial patch was used for the intra-atrial baffle, which was trimmed into a trouser-shaped patch [4]. (It was an unplanned reoperation and the condition of autologous pericardium was inadequate, so its use was abandoned.) The patch was attached with running 7–0 monofilament sutures. Continuous suture using the patch was lying from the front of the right-sided upper pulmonary vein and rerouted SVC, hepatic vein, and CS to the tricuspid valve. Hepatic vein passage was expanded with an additional patch to prevent stenosis. After the intra-atrial switch procedure, the left-sided atrium was closed (Fig. 2). Overall, CPB weaning proceeded smoothly; however, direct current cardioversion was performed for junctional ectopic tachycardia (JET). The CPB time was 99 min, and the aortic cross-clamping time was 77 min. We decided to delay sternal closure to deal with potential arrhythmias and low-output syndrome. The postoperative course was uneventful, and postoperative SpO_2_ was much improved (approximately 99–100%). Discharge echocardiography taken after Mustard operation showed that the systemic venous flow routed to the right-sided atrium(r-A) through the systemic venous baffle, whereas the pulmonary venous flow was routed to l-A through the pulmonary venous baffle. And this echocardiography showed normal biventricular function and an intact atrial baffle with no venoatrial or atrioventricular obstruction (Fig. 3). Accordingly, the patient underwent the Mustard procedure and clinical improvement was obtained.

## Discussion

In 1966, Van Praagh proposed the term IVI to refer to the rare cardiac morphology of ventricular inversion without TGA [5]. Since then, there have been very few reports of radical surgery for IVI in neonates. Herein, we report a case of neonatal IVI with situs ambiguous(inversus) morphology and heart failure. The patient developed hypoxemia after PDA ligation and atrial septostomy, and eventually underwent intra-atrial baffle repair (Mustard surgery), which resulted in a favorable outcome.

The present case suggested important feature of this disease. Existence of VSD or PDA greatly influence its clinical course.

First, in this case, VSD, which is often associated with this disease, was not observed. Lack of interventricular mixing resulted in poor oxygenation.

Arciprete and colleagues report cases of IVI with VSD (PDA absent in all cases) in which radical surgery was performed after the neonatal period. They also report a case of radical surgery being required for a newborn with IVI without VSD (surgically undetectable) [6]. Furthermore, when PDA co-exists with VSD, early intervention due to the high pulmonary blood flow is required [7, 8]. Thus, radical neonatal surgical intervention may be less necessary when PDA is absent and VSD exists. In addition, IVI often coexists with left atrial isomerism.

Left atrial isomerism is associated with multiple systemic and pulmonary venous abnormalities. Partial anomalous pulmonary venous connection or total anomalous pulmonary venous connection may increase oxygen levels in these patients [3].

Second, the patient in this case had PDA-dependent hemodynamics. We think that this is an important point that influenced our treatment strategy. In the present case, heart failure developed gradually from the age of 5 days. The cause was thought to be high pulmonary blood flow due to PDA, and we found that the PDA-dependent circulation was difficult to manage. Therefore, we performed PDA ligation and atrial septostomy, but it was difficult to achieve adequate atrial mixing as in type I TGA. Although Mauri and colleagues described that atrial septostomy was effective for the cyanosis of IVI [9], it may not always lead to improvement of oxygenation. Moreover, a ridge-like structure was observed immediately above the CS by ultrasonography during the course, indicating the anatomical feature that was likely guiding the pulmonary vein blood flow toward the tricuspid valve.

If the PDA had not been closed in this case, oxygenation could have been maintained, but heart failure would have progressed. If VSD is present, a strategy to close the PDA may be appropriate. Thus, closure of the PDA favors heart failure, but adversely affects the oxygenation level.

Another important point of this case was actual diagnosis of IVI. Because this case was diagnosed as polysplenia, it seemed to be difficult to give precise diagnosis of atrioventricular discordance. Although some reports have excluded cases of atrial situs ambiguous, others have included cases that were not defined as atrial situs solitus. Further, interestingly, all cases without ventricular septal defect and atrioventricular septal defect in the past reports of IVI showed an azygos or a hemi azygos connection [3]. In this disease, many cases have shown left isomerism and azygos or hemiazygos connection. Further, it may be difficult to distinguish the right and left atrium morphologically. In fact, defining atrial situs solitus does not seem easy. These issues are controversial among cardiac morphologists [10]. Further embryological and anatomical research is warranted in the future.

To determine the optimal treatment strategy for IVI in the neonatal period, it is necessary to consider the degree of heart failure and hypoxemia after appropriately evaluating any coexisting malformations (especially VSD and PDA). Although radical surgery by an atrial switch procedure is feasible in the neonatal period, as in the present case, the difficulty of radical surgery is thought to decrease with the physical growth of the patient. Careful follow-up with attention to potential venous stenosis in the atrium and arrhythmias in the long term is necessary in these patients.

## Conclusion

In conclusion, we successfully performed an intra-atrial switch procedure for isolated ventricular inversion in a neonate. Long-term follow-up will be necessary to ensure the maintenance of optimal cardiac function.

## Data Availability

Data sharing is not applicable to this article as no datasets were generated or analyzed during the current study.

## Abbreviations

IVI: Isolated ventricular inversion
VSD: Ventricular septal defect
SpO2: Oxygen saturation
ASD: Atrial septal defect
PDA: Patent ductus arteriosus
IVC: Inferior vena cava
SVC: Superior vena cava
CT: Computed tomography
CPB: Cardiopulmonary bypass
CS: Coronary sinus
JET: Junctional ectopic tachycardia
TGA: Transposition of the great artery
r-A: Right-sided atrium
l-A: Left-sided atrium
